# Effects of total gonadotropin dose on embryo quality and clinical outcomes with AMH stratification in IVF cycles: a retrospective analysis of 12,588 patients

**DOI:** 10.1186/s40001-024-01768-w

**Published:** 2024-03-12

**Authors:** Xiaoxue Shen, Yuhan Guo, Yan Liu, Wenyan Song, Gang Li, Haixia Jin

**Affiliations:** 1 Center for Reproductive Medicine, The First Affiliated Hospital of Zhengzhou University, Zhengzhou, 450052 Henan Province People’s Republic of China; 2 Henan Key Laboratory of Reproduction and Genetics, The First Affiliated Hospital of Zhengzhou University, Zhengzhou, China; 3 Henan Provincial Obstetrical and Gynecological Diseases (Reproductive Medicine) Clinical Research Center, The First Affiliated Hospital of Zhengzhou University, Zhengzhou, China; 4 Henan Engineering Laboratory of Preimplantation Genetic Diagnosis and Screening, The First Affiliated Hospital of Zhengzhou University, Zhengzhou, China

**Keywords:** Gonadotropin, Anti-Müllerian hormone, Embryo quality, Clinical outcomes, In vitro fertilization

## Abstract

**Background:**

Recent studies about the effect of gonadotropin (Gn) dose on the clinical outcomes of IVF are still controversial, and no studies have analyzed the relationship between Gn dose and embryo quality. Since AMH is a strong predictor of oocyte quality, we aim to evaluate the relationship between total Gn dose and embryo quality and clinical outcomes at different AMH levels in IVF cycles.

**Methods:**

A total of 12,588 patients were enrolled in the retrospective study. The included cycles were categorized by serum AMH levels (AMH ≤ 1 ng/ml, 1 ng/ml < AMH ≤ 3 ng/ml, 3 ng/ml < AMH ≤ 5 ng/ml, AMH > 5 ng/ml), total Gn dosage (< 1875 IU, 1875–3750 IU and ≥ 3750 IU) and female age (< 35 years and 35–42 years). The embryo quality and clinical outcomes were the measure outcomes.

**Results:**

The top-day3 embryos rate decreased with the increase of total Gn dose in nearly all age and AMH subgroups, but this trend was not obvious in the AMH > 5 ng/ml group and AMH ≤ 1 ng/ml group. The blastocyst formation rate and high-quality blastulation rate had a negative relationship with Gn does for women aged < 35 years in the AMH ≤ 5 ng/ml groups, except for the AMH > 5 ng/ml group (*P* < 0.001). However, when women were 35–42 years old, regardless of AMH levels, the blastocyst formation rate and high-quality blastulation rate decreased as Gn dose increased. Clinical outcomes (implantation rate, clinical pregnancy rate and live birth rate) decreased with the increase of Gn dose in all ages and AMH stratifications.

**Conclusions:**

The total dose of Gn may have different effects on embryo quality at different serum AMH levels, and the negative effects of total dose of Gn on clinical outcomes may be realized by impairing both embryo quality and endometrium.

**Supplementary Information:**

The online version contains supplementary material available at 10.1186/s40001-024-01768-w.

## Introduction

Controlled ovarian stimulation (COS) by administering exogenous gonadotropin (Gn) is one of the pivotal steps during in vitro fertilization-embryo transfer (IVF-ET) technology. Previous studies have demonstrated that increasing the dose of Gn can increase the number of oocytes retrieved, thus enhancing the cumulative live birth rate [1–4]. However, the risk of ovarian hyperstimulation syndrome (OHSS) will also be raised by high dose of Gn [5]. Although patients suffering from OHSS can choose frozen-thawed embryo transfer to eliminate the adverse impact on the clinical outcomes [6], serious harm to maternal physiology caused by potentially life-threatening complications of OHSS still cannot be ignored.

How to use Gn safely is getting more and more attention, and many studies are focusing on the impact of clinical outcomes. A retrospective analysis of 650000 IVF-ET cycles revealed that Gn dose was negatively correlated with live birth rate [7]. However, Yang et al. indicated that live birth rate increased with Gn dose in normal responders [8]. And in preimplantation genetic testing (PGT) cycles, high doses of Gn did not affect clinical pregnancy rate and live birth rate [9, 10].

In addition to potentially affecting clinical outcomes, high doses of Gn may affect embryo quality as well. Most of the effects of exogenous Gn on oocytes or embryo quality are concentrated on animal experiments, and high follicle stimulating hormone (FSH) dose has been confirmed to have adverse effects on murine oocytes and embryos [11–14]. Some studies have indicated that high doses of exogenous Gn may increase aneuploidy by affecting chromosome segregation in normal meiosis of embryos [15, 16]. However, recent studies have refuted these claims, showing that high doses of Gn do not increase aneuploidy in PGT cycles [9, 10, 17, 18]. Notably, there have been few studies on the effect of high doses of exogenous Gn on embryo quality in IVF/ intracytoplasmic sperm injection (ICSI) cycles rather than PGT cycles.

Currently, ovarian stimulation protocols are flexibly administered according to the characteristics of infertile couples to safely and effectively enhance clinical outcomes. The time and dose of Gn vary among different ovarian stimulation protocols, such as GnRH agonist and GnRH antagonist protocols. Although the same ovarian stimulation protocol is applied, clinicians use different doses and duration of Gn depending on the female age, body mass index (BMI), ovarian reserve, and other indices that affect embryo quality and clinical outcomes [19, 20]. Anti-Müllerian hormone (AMH) is a strong marker of ovarian reserve and ovarian response [21]. Given that Gn dose has controversial effects on oocyte quality and clinical outcomes in many studies, we designed a retrospectively and AMH-stratified study to explore the effects of Gn dose on embryo quality and clinical outcomes among tubal factor infertility couples with follicular phase GnRH agonist protocol.

## Materials and methods

### Patients and study design

A retrospective analysis was performed on infertile patients who underwent IVF from 2017 to 2020 at our reproductive medicine center. A total of 12,588 cycles fulfilled the inclusion criteria, which were ①fresh autologous first IVF-ET cycles, ②tubal factor infertility, ③follicular phase GnRH agonist protocol, and ④female BMI < 25 kg/m^2^. The following cycles were excluded: ①women aged > 42 years old; ②PGT cycles; ③testicular epididymal sperm aspiration or percutaneous epididymal sperm aspiration cycles; ④no oocytes retrieved cycles; ⑤women with other diseases.

The included cycles were categorized by serum AMH levels (AMH ≤ 1 ng/ml, 1 ng/ml < AMH ≤ 3 ng/ml, 3 ng/ml < AMH ≤ 5 ng/ml, AMH > 5 ng /ml), and total Gn dosage (group A: < 1875 IU, group B: 1875–3750 IU and group C: ≥ 3750 IU) and female age (< 35 years and 35–42 years) based on clinical practice experience of our center. When the clinical outcomes (implantation rate, clinical pregnancy rate and live birth rate) of different Gn doses were observed at the same age and serum AMH levels category, we included the fresh cycles (6788 cycles) in which two embryos at the cleavage stage were transferred and endometrial thickness was ≥ 7 mm on ET day. The study was approved by the Ethics Committee of the First Affiliated Hospital of Zhengzhou University (Reference: 2021-KY-0223-001).

### IVF procedures

The standard operating procedure of follicular phase GnRH agonist protocol in our center is as follows. A prolonged action GnRH agonist (3.75 mg, IPSEN, France) was given on the 2nd–3rd day of menstruation. After 30–42 days, when the patients met the criteria for down-regulation (no follicles > 10 mm in diameter; estradiol < 183 pmol/L, luteinizing hormone (LH) < 3 IU/L), the initial Gn dose was selected based on female age, BMI, AMH and ovarian response to previous COS. During COS, Gn dose was adjusted according to hormone levels and follicular size detected by ultrasound. The patients were all treated with the same Gn (gonal-f, Merck), but the choice of whether to use human menopausal gonadotropin was based on the size and growth of the follicles in each patient. When the following criteria were met: one dominant follicle diameter ≥ 20 mm, three follicles diameter ≥ 17 mm, or 2/3 follicles diameter ≥ 16 mm, 2000 IU of human chorionic gonadotropin (hCG) (Livzon, China) and 250 µg of recombinant hCG (Merck, Italy) were administered.

A 37-h period after the injection of hCG, the follicles were punctured with transvaginal ultrasound. The isolated oocytes were then fertilized in vitro with IVF or ICSI, depending on semen quality. The Perter scoring system [22] was used to evaluate the morphology of the cleavage embryos on day3 and Gardner scoring criteria [23] were used to evaluate embryos at the blastocyst stage. According to the observation of embryo morphology on day3, the embryos were cryopreserved, transferred, or cultured until day5/6 and the remaining embryos were discarded. Embryos arranged for blastocyst cultured were incubated at 37 ℃, 6% CO_2_, and 5% O_2_ on day5 or day6. We defined all embryos on day3 having a score of I or II as top-day3 embryos, and embryos on Day5/6 having a score of ≥ 3BB as high-quality blastocysts. All patients were given the same luteal support on the day of oocyte retrieved until 65 days after ET.

### Outcome measures

The embryo quality parameters included the top-day3 embryo rate, blastocyst formation rate and high-quality blastulation rate. The top-day3 embryo rate was calculated by dividing the top-day3 embryos number by the two distinct pronuclei (2PN) zygote number. The blastocyst formation rate was the ratio of blastocyst number divided by the number of embryos on day 3 to extend culture. The high-quality blastulation rate was calculated by dividing the high-quality blastocyst number by the number of embryos on day 3 to extend culture. The clinical outcomes included the implantation rate, clinical pregnancy rate and live birth rate. The implantation rate was calculated as the number of gestational sac detected via transvaginal ultrasound divided by the number of embryos transferred. Visualization of one or more gestational sacs via transvaginal ultrasound between 6 and 7 gestational weeks was regarded as clinical pregnancy. The clinical pregnancy rate was calculated by dividing the number of clinical pregnancy cycles by the embryo transferred cycles. The live birth rate was calculated by dividing the number of live birth cycles by the embryo transferred cycles.

### Measurement of AMH

Venous blood (4 mL) was collected from the patients on the 2nd to 4th day of the menstrual cycle and loaded into a procoagulant tube containing inert separation gel. After centrifugation at 3000r/min for 10 min within 2 h, serum was separated, and then AMH was measured by biochemical analyzer (Roche Cobase411). The relevant detection kits and instruments were provided by Roche, Germany, and were operated in strict accordance with standard procedures.

### Statistical analysis

Continuous variables were expressed as mean ± SD. They were compared using the one-way ANOVA test or the Kruskal–Wallis non-parametric test. Categorical variables were expressed as n (%) and compared with the Chi-square test and Fishers exact test. Multivariable regression analysis was performed to evaluate effect of total Gn dose on clinical pregnancy rate and live birth rate. Covariates included female age, serum AMH levels, Lg10 (total Gn dosage), the stage of ET, number of oocytes retrieved, number of ET and endometrial thickness on ET day. *P* < 0.05 was considered statistically significant. All statistical analyses were conducted in SPSS 21.0.

## Results

**Fig. 1:**
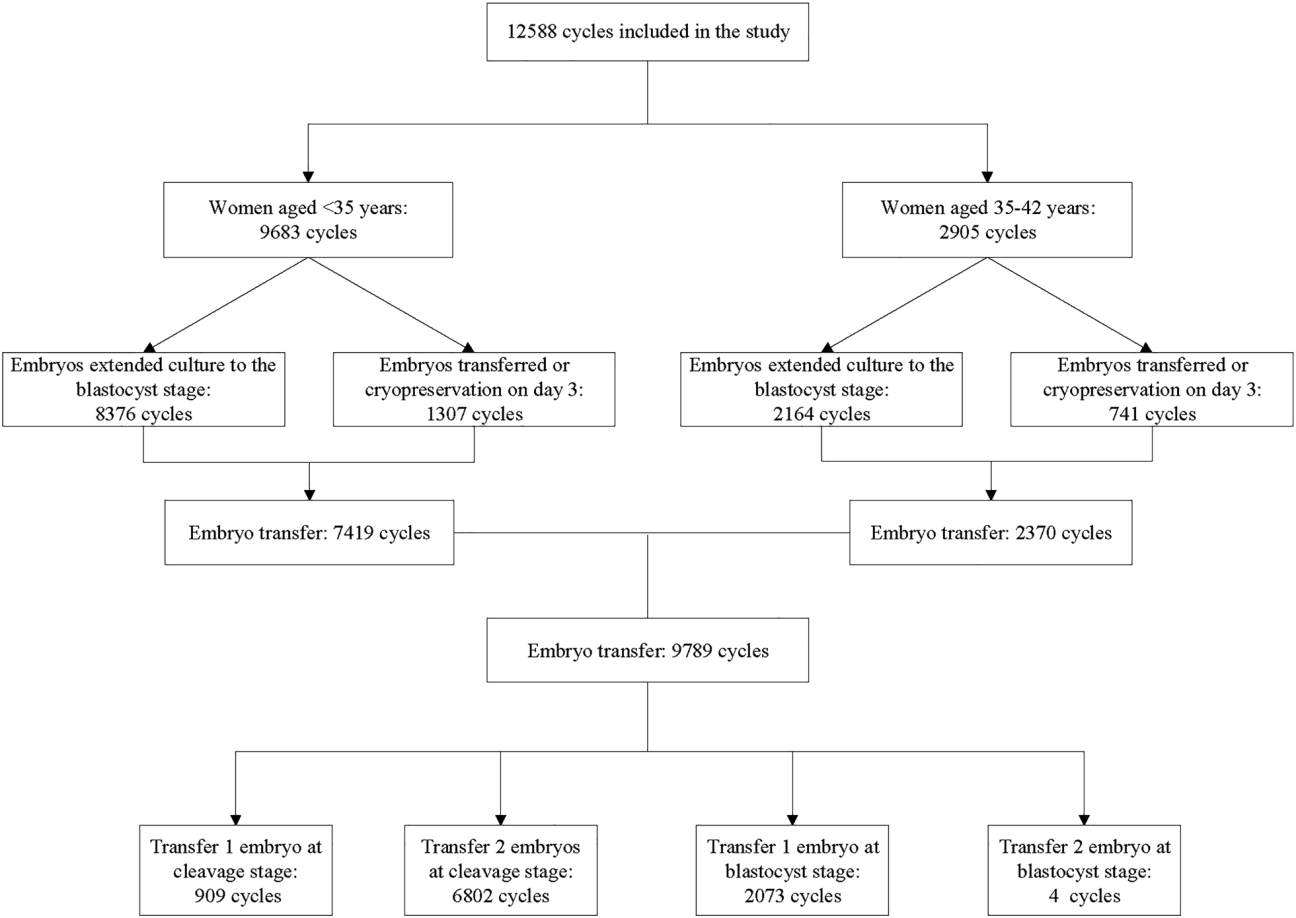
Flowchart showing data selection process and cycle distribution

A total of 12,588 cycles were analyzed for the effects of Gn dosage on embryo quality and clinical outcomes. The distribution of blastocyst culture and embryo transfer cycles is shown in Fig. 1. There were 9683 cycles for women aged < 35 years and 2905 cycles for women aged between 35 and 42 years, and baseline characteristics with age groups are included in Additional file 1: Table S1. As shown in Table 1, the demographic characteristics of the population are summarized. Female age, BMI, Gn duration and basal FSH levels increased with Gn dose. However, there is a tendency to decrease serum AMH levels and the number of oocytes retrieved with increasing total Gn dosage. Interestingly, there was a significant difference (*P* < 0.001) in embryo quality and clinical outcomes in different Gn dose groups.

**Table 1:** Cycle characteristics of different Gn doses among the study population

Total Gn dosage(IU)	< 1875	1875–3750	≥ 3750	*P* Value
Cycles	4347	6458	1783	
Female age (years)	29.18 ± 3.67	31.33 ± 4.47	34.35 ± 4.68	< 0.001
Female BMI (kg/m^2^)	20.90 ± 1.95	21.70 ± 1.94	21.86 ± 1.84	< 0.001
Serum AMH (ng/ml)	4.50 ± 1.82	2.81 ± 1.64	1.29 ± 0.77	< 0.001
Basal FSH (mIU/ml)	6.18 ± 1.45	7.09 ± 2.08	8.70 ± 3.50	< 0.001
Gn duration (days)	12.30 ± 1.45	13.41 ± 1.79	15.18 ± 1.93	< 0.001
Number of oocytes retrieved	16.73 ± 6.58	13.16 ± 6.22	8.03 ± 4.57	< 0.001
Number of MII	13.83 ± 5.95	10.68 ± 5.45	6.61 ± 4.09	< 0.001
Normal fertilization rate (%)	65.82	64.34	64.27	< 0.001
Average number of ET	1.62 ± 0.49	1.72 ± 0.45	1.75 ± 0.44	< 0.001
Stage of ET				
Day 3 (%)	67.13	81.55	93.06	< 0.001
Day 5/6 (%)	32.87	18.45	6.94	< 0.001
Endometrial thickness on ET day	12.64 ± 2.63	12.42 ± 2.74	11.99 ± 2.73	< 0.001
Embryo quality				
Top-day3 embryo rate (%)	70.67	67.34	66.03	< 0.001
Blastocyst formation rate (%)	60.27	53.76	43.04	< 0.001
High-quality blastulation rate (%)	22.20	18.09	12.18	< 0.001
Clinical outcome				
Implantation rate (%)	57.27	45.46	31.70	< 0.001
Clinical pregnancy rate (%)	72.72	61.89	46.31	< 0.001
Live birth rate (%)	57.08	47.64	34.12	< 0.001

*AMH* anti-Müllerian hormone, *Gn* gonadotropin, *BMI* body mass index, *FSH* follicle-stimulating hormone, *MII* mature oocytes, *ET* embryo transfer. *P* < 0.05 indicates significant difference among the three Gn groups

### The effect of total Gn dose on embryo quality

**Fig. 2:**
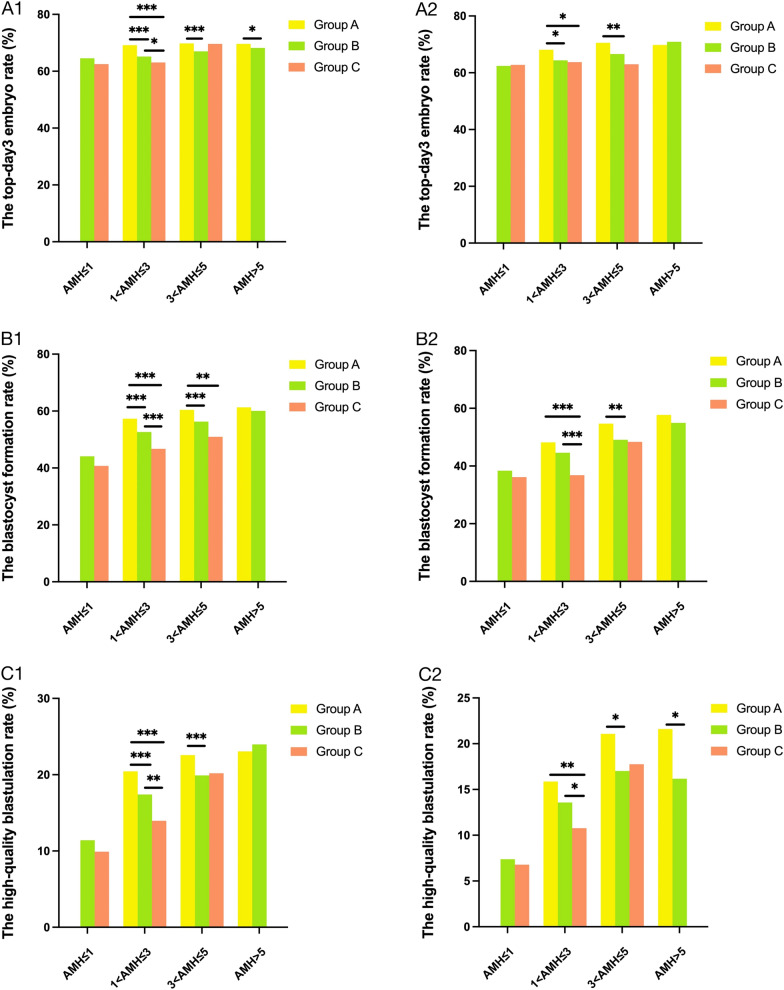
The embryo quality of different total Gn dosage groups with AMH stratification.** A1**, ** B1**, ** C1**: female age < 35 years; **A2**, ** B2**, ** C2**: female aged 35–42 years. *AMH* anti-Müllerian hormone, *Gn* gonadotropin. Group A: < 1875 IU, Group B: 1875–3750 IU, Group C: ≥ 3750 IU. *P < 0.05, **P < 0.01, ***P < 0.001

Table 2 and Fig. 2 show the relationship between Gn dose and embryo quality. Across nearly all AMH subgroups for women aged < 35 years, except for in group C with 3-5 ng/ml AMH levels, the top-day3 embryo rates in low-dose Gn groups were higher than in high-dose Gn groups. And in the 35–42 years group, the top-day3 embryo rates in low-dose Gn groups were also higher than in high-dose groups across nearly all AMH subgroups, except for in group C with AMH ≤ 1 ng/ml and in group B with AMH > 5 ng/ml. The difference was statistically significant when the three Gn groups were compared with each other for women aged < 35 years with 1–3 ng/ml AMH levels (*P* < 0.05). The top-day3 embryo rate in group A was significantly higher than in group B (*P* < 0.05) when women were younger than 35 years old with AMH > 3 ng/ml. There was no significant difference in the top-day3 embryo rate for women aged < 35 years with 3-5 ng/ml AMH levels between group C and group A or group B, respectively (*P* > 0.05). The same observation was also generally held for women aged 35–42 years with 3-5 ng/ml AMH levels (*P* < 0.05). Group A had the highest top-day3 embryo rate compared to the other Gn groups in the 1–3 ng/ml AMH group and 35–42 years group (*P* < 0.05).

**Table 2:** The embryo quality of different total Gn dosage groups with AMH stratification

AMH levels	Total Gn dosage (IU)			*P* value		
	< 1875 (A)	1875–3750 (B)	≥ 3750 (C)	*P*_AB_	*P*_AC_	*P*_BC_
The top-day3 embryo rate (%)						
Age < 35 Y						
AMH ≤ 1 ng/ml	0	64.54 (1307/2025)	62.51 (1154/1846)	–	–	0.192
1 < AMH ≤ 3 ng/ml	69.16 (5449/7879)	65.16(12,975/19913)	63.07 (1433/2272)	< 0.001	< 0.001	0.049
3 < AMH ≤ 5 ng/ml	69.82 (12,360/17702)	67.01 (8224/12272)	69.61 (268/385)	< 0.001	0.955	0.296
AMH > 5 ng/ml	69.61 (10,935/15710)	68.20 (4383/6427)	0	0.040	–	–
Age 35–42 Y						
AMH ≤ 1 ng/ml	0	62.43 (525/841)	62.77 (1182/1883)	–	–	0.864
1 < AMH ≤ 3 ng/ml	68.09 (638/937)	64.37 (4029/6259)	63.76 (1443/2263)	0.028	0.020	0.609
3 < AMH ≤ 5 ng/ml	70.56 (1299/1841)	66.60 (1892/2841)	62.99 (97/154)	0.005	0.055	0.381
AMH > 5 ng/ml	69.82 (835/1196)	70.89 (699/986)	0	0.605	–	–
The blastocyst formation rate (%)						
Age < 35 Y						
AMH ≤ 1 ng/ml	0	44.10 (340/771)	40.73 (255/626)	–	–	0.211
1 < AMH ≤ 3 ng/ml	57.32 (2423/4227)	52.65 (5023/9540)	46.73 (472/1010)	< 0.001	< 0.001	< 0.001
3 < AMH ≤ 5 ng/ml	60.40 (6091/10084)	56.27 (3806/6764)	50.96 (106/208)	< 0.001	0.006	0.136
AMH > 5 ng/ml	61.29 (5841/9530)	60.07 (2278/3792)	0	0.194	–	–
Age 35–42 Y						
AMH ≤ 1 ng/ml	0	38.38 (104/271)	36.14 (219/606)	–	–	0.545
1 < AMH ≤ 3 ng/ml	48.21 (243/504)	44.62 (1285/2880)	36.81 (325/883)	0.146	< 0.001	< 0.001
3 < AMH ≤ 5 ng/ml	54.68 (532/973)	49.09 (704/1434)	48.39 (30/62)	0.008	0.359	1.000
AMH > 5 ng/ml	57.72 (430/745)	54.95 (333/606)	0	0.321	–	–
The high-quality blastulation rate (%)						
Age < 35 Y						
AMH ≤ 1 ng/ml	0	11.41 (88/771)	9.90 (62/626)	–	–	0.386
1 < AMH ≤ 3 ng/ml	20.44 (864/4227)	17.40 (1660/9540)	13.96 (141/1010)	< 0.001	< 0.001	0.006
3 < AMH ≤ 5 ng/ml	22.58 (2277/10084)	19.90 (1346/6764)	20.19 (42/208)	< 0.001	0.451	0.930
AMH > 5 ng/ml	23.05 (2197/9530)	23.97 (909/3792)	0	0.266	–	–
Age 35–42 Y						
AMH ≤ 1 ng/ml	0	7.38 (20/271)	6.77 (41/606)	–	–	0.774
1 < AMH ≤ 3 ng/ml	15.87 (80/504)	13.58 (391/2880)	10.76 (95/883)	0.185	0.007	0.029
3 < AMH ≤ 5 ng/ml	21.07 (205/973)	17.02 (244/1434)	17.74 (11/62)	0.014	0.630	1.000
AMH > 5 ng/ml	21.61 (161/745)	16.17 (98/606)	0	0.012	–	–

*P*_AB_: < 1875 IU group vs. 1875–3750 IU group. *P*_AC_: < 1875 IU group vs. ≥ 3750 IU group. *P*_BC_: 1875–3750 IU group vs. ≥ 3750 IU group. *AMH* anti-Müllerian hormone, *Gn* gonadotropin. *P* < 0.05 was considered statistically significant. -, not applicable

The blastocyst formation rates were inversely correlated with total Gn dose in all AMH and age groups. In the < 35 years group, the difference was statistically significant across all Gn groups with 1–3 ng/ml AMH group (*P* < 0.001). Moreover, the blastocyst formation rate of group A was statistically significant compared with other Gn groups in < 35 years group within 3–5 ng/ml AMH (*P* < 0.05). There was no statistically significant difference in blastocyst formation rate between group A and group B in < 35 years group within AMH > 5 ng/ml (*P* > 0.05). In the 35–42 years group, the blastocyst formation rate of group C was significantly lower than that of other two Gn groups within 1-3 ng/ml AMH (*P* < 0.001), and group A was significantly higher than group B within 3–5 ng/ml AMH(*P* < 0.001). Although the negative correlation between blastocyst formation rate and Gn dose was observed in other groups, the difference did not reach statistical significance (*P* > 0.05). Overall, the blastocyst formation rates in the high-dose Gn group were lower than that in the low-dose Gn group.

The rate of high-quality blastulation decreased with the increase of Gn dose in nearly all AMH and age categories apart form in group B for women aged < 35 years with AMH > 5 ng/ml. The difference in high-quality blastulation rate was statistically significant when the three Gn groups were compared with each other for women aged < 35 years within 1–3 ng/ml group (*P* < 0.01). There was no significant difference in the high-quality blastulation rate in < 35 years group within 3-5 ng/ml AMH between group C and either group A or group B (*P* > 0.05). Differences were significant at *P* < 0.05 when group A was compared with group B for women aged < 35 years with 3–5 ng/ml AMH and for women aged 35–42 years with AMH > 3 ng/ml. The high-quality blastulation rate in group C was significantly lower than that in the other two Gn groups, which was observed in the 35–42 years group with 1–3 ng/ml AMH (*P* < 0.05).

### The relationship between Gn dose and clinical outcomes

**Fig. 3:**
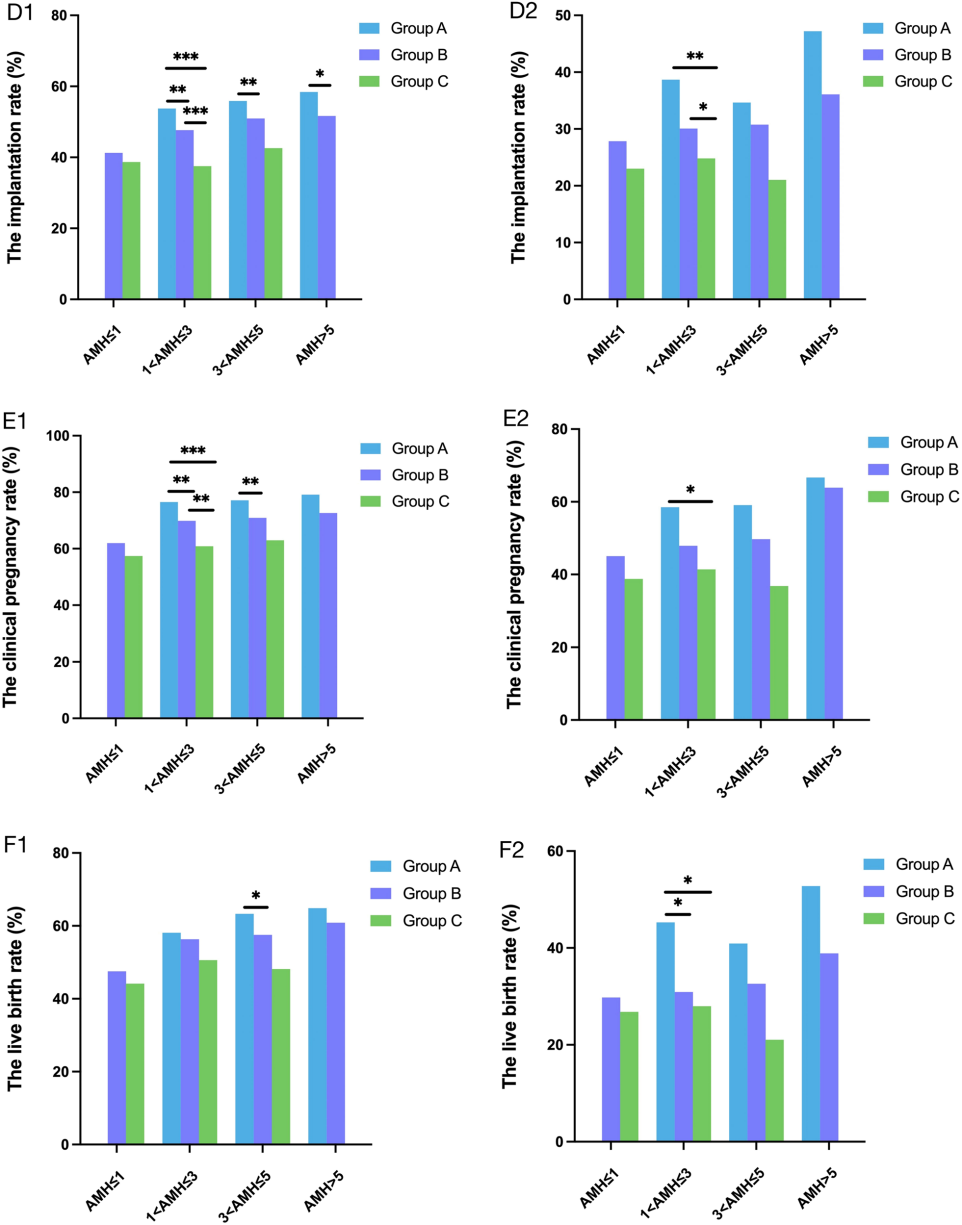
The clinical outcome of different total Gn dosage groups with AMH stratification. The IVF-ET cycles of 2 embryos at the cleavage stage with endometrial thickness ≥ 7 mm on ET day were included. **D1**, **E1**, **F1**: female age < 35 years; **D2**, **E2**, **F2**: female aged 35–42 years. *AMH* anti-Müllerian hormone, *Gn* gonadotropin. Group A: < 1875 IU, Group B: 1875–3750 IU, Group C: ≥ 3750 IU. *P < 0.05, **P < 0.01, ***P < 0.001

Logistic regression models (Table 3) showed clinical pregnancy rate was negatively correlated with female age (OR = 0.930, 95% CI 0.920–0.939, *P* < 0.001), Gn dosage (OR = 0.270, 95% CI 0.193–0.378, *P* < 0.001), number of oocytes retrieved (OR = 0.989, 95% CI 0.979–0.998, *P* = 0.021) and stage of ET (day 3) (OR = 0.592, 95% CI 0.493–0.710, *P* < 0.001). The clinical pregnancy rate was positively correlated with AMH (OR = 1.053, 95% CI 1.020–1.087, *P* = 0.001), number of ET (OR = 2.143, 95% CI 1.849–2.484, *P* < 0.001) and endometrial thickness (OR = 1.058, 95% CI 1.042–1.076, *P* < 0.001). Similarly, live birth rate was negatively correlated with female age (OR = 0.922, 95% CI 0.913–0.931, *P* < 0.001), Gn dosage (OR = 0.448, 95% CI 0.327–0.615, *P* < 0.001) and stage of ET (day 3) (OR = 0.721, 95% CI 0.598–0.868, *P* = 0.001). The live birth rate was positively correlated with AMH (OR = 1.050, 95% CI 1.019–1.081, *P* = 0.001), number of ET (OR = 1.899, 95% CI 1.626–2.217, *P* < 0.001) and endometrial thickness (OR = 1.064, 95% CI 1.048–1.081, *P* < 0.001).Table 4 and Fig. 3 show the relationship between total Gn dose and clinical outcomes with AMH stratification in IVF-ET cycles where two embryos at the cleavage stage were transferred and endometrial thickness was ≥ 7 mm on ET day. Implantation rate decreased with increasing Gn dose across all female age and AMH categories. Differences were significant at *P* ≤ 0.001 across all Gn groups for women aged < 35 years with 1–3 ng/ml AMH levels. Similarly, differences were significant at *P* < 0.05 when comparing group A and B for women aged < 35 years with > 3 ng/ml AMH levels. The implantation rate of group C was significantly lower than that of the other two Gn groups within 35–42 years group and 1–3 ng/ml AMH levels (*P* < 0.05). There was no statistically significant difference in implantation rate between group B and group C (*P* > 0.05).

**Table 3:** Multiple logistic regression analysis of clinical pregnancy rate or live birth rate in IVF/ICSI cycles

Variables	Clinical pregnancy rate			Live birth rate		
	Coeff	OR (95% CI)	*P* Value	Coeff	OR (95% CI)	*P* Value
Female age	−0.073	0.930 (0.920–0.939)	< 0.001	− 0.081	0.922 (0.913–0.931)	< 0.001
AMH	0.052	1.053 (1.020–1.087)	0.001	0.049	1.050 (1.019–1.081)	0.001
Lg10 (Gn dosage)	−1.308	0.270 (0.193–0.378)	< 0.001	− 0.803	0.448 (0.327–0.615)	< 0.001
Number of oocytes retrieved	−0.011	0.989 (0.979–0.998)	0.021	−0.005	0.995 (0.986–1.004)	0.302
Stage of ET ( day3)	−0.525	0.592 (0.493–0.710)	< 0.001	−0.328	0.721 (0.598–0.868)	0.001
Number of ET	0.762	2.143 (1.849–2.484)	< 0.001	0.641	1.899 (1.626–2.217)	< 0.001
Endometrial thickness on ET day	0.057	1.058 (1.042–1.076)	< 0.001	0.062	1.064 (1.048–1.081)	< 0.001

*AMH* anti-Müllerian hormone, *Gn* gonadotropin, *ET* embryo transfer, *Coeff* coefficient, *OR* odds ratio, *95% CI* 95% confidence interval

**Table 4:** The clinical outcome of different total Gn dosage groups with AMH stratification

AMH levels	Total Gn dosage (IU)			*P* value		
	< 1875 (A)	1875–3750 (B)	≥ 3750 (C)	*P*_AB_	*P*_AC_	*P*_BC_
The implantation rate (%)						
Age < 35 Y						
AMH ≤ 1 ng/ml	0	41.25 (217/526)	38.67 (198/512)	–	–	0.410
1 < AMH ≤ 3 ng/ml	53.74 (503/936)	47.66 (1629/3418)	37.55 (190/506)	0.001	< 0.001	< 0.001
3 < AMH ≤ 5 ng/ml	55.87 (856/1532)	50.94 (708/1390)	42.59 (23/54)	0.008	0.069	0.267
AMH > 5 ng/ml	58.43 (582/996)	51.65 (219/424)	0	0.019	–	–
Age 35–42 Y						
AMH ≤ 1 ng/ml	0	27.86 (73/262)	23.01 (127/552)	–	–	0.139
1 < AMH ≤ 3 ng/ml	38.68 (41/106)	30.07 (329/1094)	24.81 (133/536)	0.078	0.004	0.030
3 < AMH ≤ 5 ng/ml	34.66 (61/176)	30.75 (115/374)	21.05 (8/38)	0.379	0.127	0.266
AMH > 5 ng/ml	47.22 (34/72)	36.11 (26/72)	0	0.237	–	–
The clinical pregnancy rate (%)						
Age < 35 Y						
AMH ≤ 1 ng/ml	0	61.98 (163/263)	57.42 (147/256)	–	–	0.325
1 < AMH ≤ 3 ng/ml	76.50 (358/468)	69.87 (1194/1709)	60.87 (154/253)	0.006	< 0.001	0.005
3 < AMH ≤ 5 ng/ml	77.15 (591/766)	70.94 (493/695)	62.96 (17/27)	0.007	0.104	0.392
AMH > 5 ng/ml	79.12 (394/498)	72.64 (154/212)	0	0.064	–	–
Age 35–42 Y						
AMH ≤ 1 ng/ml	0	45.04 (59/131)	38.77 (107/276)	–	–	0.237
1 < AMH ≤ 3 ng/ml	58.49 (31/53)	47.90 (262/547)	41.42 (111/268)	0.152	0.024	0.086
3 < AMH ≤ 5 ng/ml	59.09 (52/88)	49.73 (93/187)	36.84 (7/19)	0.156	0.126	0.340
AMH > 5 ng/ml	66.67 (24/36)	63.89 (23/36)	0	1.000	–	–
The live birth rate (%)						
Age < 35 Y						
AMH ≤ 1 ng/ml	0	47.53 (125/263)	44.14 (113/256)	–	–	0.481
1 < AMH ≤ 3 ng/ml	58.12 (272/468)	56.35 (963/1709)	50.59 (128/253)	0.494	0.059	0.090
3 < AMH ≤ 5 ng/ml	63.32 (485/766)	57.55 (400/695)	48.15 (13/27)	0.028	0.155	0.428
AMH > 5 ng/ml	64.86 (323/498)	60.85 (129/212)	0	0.348	–	–
Age 35–42 Y						
AMH ≤ 1 ng/ml	0	29.77 (39/131)	26.81 (74/276)	–	–	0.555
1 < AMH ≤ 3 ng/ml	45.28 (24/53)	30.90 (169/547)	27.99 (75/268)	0.044	0.015	0.416
3 < AMH ≤ 5 ng/ml	40.91 (36/88)	32.62 (61/187)	21.05 (4/19)	0.223	0.123	0.438
AMH > 5 ng/ml	52.78 (19/36)	38.89 (14/36)	0	0.344	–	–

The IVF-ET cycles of 2 embryos at the cleavage stage with endometrial thickness ≥ 7 mm on ET day were included. *P*_AB_: < 1875 IU group vs. 1875–3750 IU group. *P*_AC_: < 1875 IU group vs. ≥ 3750 IU group. *P*_BC_: 1875–3750 IU group vs. ≥ 3750 IU group. *AMH* anti-Müllerian hormone, *Gn* gonadotropin. *P* < 0.05 was considered statistically significant. -, not applicable

Interestingly, the clinical pregnancy rate decreased as Gn dose increased in all ages and AMH stratifications. Groups A, B, and C were significantly different (*P* < 0.05) when pairwise compared to each other for women aged < 35 years with 1–3 ng/ml AMH levels. There was a significant difference (*P* < 0.05) between group A and group B in < 35 years group with 3–5 ng/ml AMH levels. There was no significant difference in clinical pregnancy rate for women aged < 35 years with 3–5 ng/ml AMH levels between group C and either group A or group B (*P* > 0.05). And the clinical pregnancy rate of group A was significantly higher than that of group C within 35–42 years group and 1–3 ng/ml AMH levels (*P* < 0.05).

Although the live birth rate was also negatively correlated with Gn dose independent of female age and serum AMH levels, a little significant difference was observed. Differences were significant at *P* < 0.05 when comparing group A and B for women aged < 35 years with 3–5 ng/ml AMH levels. The live birth rate of group A was significantly lower than that of the other two Gn groups within the 35–42 years group and 1–3 ng/ml AMH levels (*P* < 0.05).

## Discussion

Clinicians perform individual ovarian stimulation based on a woman's age, ovarian reserve and other patient characteristics to reduce the incidence of OHSS [24] and improve the success of therapy. It is confirmed that the number of oocytes retrieved and ovarian response are related to AMH and AMH has higher sensitivity and specificity than FSH [19, 25]. AMH is a relatively stable hormone that does not vary with the menstrual cycle [26], and it is a reliable predictor of IVF clinical outcomes [27, 28] whose serum concentration is significantly correlated with oocyte quality [28, 29]. To minimize the confounding factors such as female age, serum AMH level and female etiology, therefore, we performed a retrospective analysis of the effects of exogenous total Gn dose on embryo quality and clinical outcomes under age and AMH stratification among women suffering from tubal factor infertility with follicular phase GnRH- agonist protocol.

Our study found that when serum AMH level was between 1 and 5 ng/ml, the top-day3 embryo rate, blastocyst formation rate and high-quality blastulation rate decreased with the increase of total dose of Gn, regardless of age. This trend is not obvious in the AMH ≤ 1 ng/ml and > 5 ng/ml subgroups. In the AMH ≤ 1 ng/ml subgroup, the blastocyst formation rate and the high-quality blastulation rate of the ≥ 3750 IU group were lower than those of 1875-3750 IU group(*P* > 0.05) with all ages, but only at < 35 years subgroup, the 1875-3750 IU group had a higher rate of top-Day3 embryos than the ≥ 3750 IU group. Interestingly, when serum AMH level was more than 5 ng/ml, Gn dose had little effect on embryo quality, especially in the < 35 years subgroup. Altogether, there is a corollary in our research: Gn dose may have different effects on embryo quality in different AMH levels.

Ovarian stimulation makes multiple follicles growth and development and increases the number of follicles recruited by eliminating the limitation of physiological mechanism for selecting single follicle and the inhibition of dominant follicle for the secondary follicle growth. Nevertheless, high dose Gn has been certified to be detrimental to animal oocyte and embryo development. Roberts et al. demonstrated that the addition of high dose FSH leads to abnormal chromosome separation of mouse oocytes in vitro [13]. And Fushii et al. found that high concentration of FSH medium could collapse part of oocyte-cumulus cell complexes, increase the number of degenerated oocytes [30]. Other studies on bovine oocyte-cumulus cell cultured in vitro illustrated that high concentration of Gn increased the apoptosis of granulosa cells, delayed the development of embryos [31] and lead to maturation arrest [32]. Superovulated mice embryos had a delay in blastocyst hatching and fetal development compared with naturally cycling mice [14]. However, a study demonstrated that Gn dose was positively correlated with oocyte maturation in normal responders with gonadotropin releasing hormone (GnRH) antagonist protocol [8]. Demirol et al. showed that there was no difference in fertilization rate between micro-dose and multi-dose protocols in ICSI cycles for poor responders [33]. At present, there are three potential mechanisms by which Gn affects oocyte quality and embryo development: i. High doses of exogenous Gn induce superphysiological estrogen concentration in vivo, and oocytes exposed to this would be impaired, then the development ability of embryos will be arrested [34]; ii. The simultaneous development of multiple follicles during ovulation induction affects the natural selection of dominant follicles and leads to the recruitment of some secondary follicles, thus affecting the quality of embryos [35]; iii. Using high exogenous Gn interferes with the meiosis of oocytes and chromosome segregation, which increases the rate of embryo aneuploidy [36]. Yet, recent studies have proved that the use of exogenous Gn does not affect the rate of embryo aneuploidy [9, 10, 37]. Depending on our results, we speculated that the quality of oocytes and the ability of oocyte resistance to superphysiological levels of estrogen concentrations are weak when serum AMH levels is relatively low, and high doses of exogenous Gn result in an increased proportion of poor quality oocytes retrieved, which diminished subsequent embryonic development capacity. In contrast, when serum AMH levels are relatively high, the secondary oocytes recruited were of relatively good quality which have the ability to resist superphysiological estrogen levels, hence it does not seem to affect the development of oocytes and embryos. Brinsden et al. found that in ICSI cycles, Serum AMH was associated with cytoplasm granulation, abnormally amorphous oocytes (*P* < 0.01), extended perivitelline space (*P* < 0.001), granulated perivitelline space (*P* < 0.05), fragmented polar body (*P* < 0.001), and average of oocyte quality index (*P* < 0.01), which seems to support our inference [27]. It is noteworthy that the high-quality blastulation rate in the < 1875 IU group is significantly higher than that in the 1875–3750 IU group (*P* < 0.05), but there was no statistical difference between the top-Day3 embryo rate and blastocyst formation rate when comparing the < 1875 IU group and the 1875–3750 IU group for women aged 35–42 years with > 5 ng/ml AMH levels. This may be due to oocyte degeneration as women age, and the difference can be perceived by the high-quality blastulation rate which is a better indicator of the embryo's developmental potential than the other two indicators.

Furthermore, we found that implantation rate, clinical pregnancy rate and live birth rate all decreased with increasing Gn dose, although small sample sizes may not have detected significant statistical differences in some subgroups. This is consistent with the conclusion of Baker L et al.’s study [7]. When comparing the < 1875 IU group and the 1875-3750 IU group for women aged < 35 years with AMH > 5 ng/ml who have almost no difference in embryo quality, the implantation rate decreased by 6.8% (*P* < 0.05), the clinical pregnancy rate decreased by 6.5% and the live birth rate decreased by 4.0% (Table 3). Although there were no statistically significant differences in the implantation rate, the clinical pregnancy rate and the live birth rate between the < 1875 IU group and the 1875–3750 IU group due to the small sample size, a trend was observed that Gn dose was negatively correlated with the implantation rate, the clinical pregnancy rate and the live birth rate. Similarly, Erika M et al. found that the live birth rate was affected by the high dose of FSH during fresh ET cycles, while the live birth rate of the subsequent paired frozen-thawed ET did not seem to be affected by the dose of FSH, suggesting that the endometrium may be negatively affected [38]. Moreover, Chang et al. confirmed high concentration of Gn inhibits endometrial cell proliferation in vitro [39], which is consistent with the change trend of endometrial thickness with Gn dose in Table 1. However, Lensen et al. did not find that the FSH dose in any particular ORT population influenced rates of live birth [40]. In summary, we believe that the high doses of Gn may have adverse effects on clinical outcomes, which are achieved through damage to both endometrium and embryo quality.

The best advantage of this study is that this is a large retrospective study and the first study to explore the effects of exogenous Gn dose on embryo quality and clinical outcomes with AMH stratification. Although this retrospective study demonstrates the effects of Gn dosage on embryo quality and clinical outcomes, the data from multiple centers may help to validate our single center study. The total dose of Gn recorded in our center included both FSH-only and human menopausal gonadotropin, so we have no direct evidence to prove whether the FSH-only dose has an adverse effect on embryo quality and clinical outcomes. As this is a retrospective study, review bias and completeness of data may affect study outcomes. Sperm characteristics are one of the important factors affecting embryo quality. Although we tried to exclude sperm factors affecting embryo quality, missing data on sperm characteristics may have an impact on the results of the study. To increase the ET opportunities of patients, our center usually chooses to ET on day 3. This not only leads to be unable to analyze blastocyst transfers cycles in this study (the samples were too small), but also leads to not all zygotes extended culture to the blastocyst stage, which may produce bias when exploring the effect of Gn dose on embryo quality. In addition, some subgroup samples were small (there were no samples in the Gn < 1875 IU group with AMH ≤ 1 ng/ml), which may cause bias in the results. At the same time, the number of GnRH-antagonist cycles that met the inclusion criteria was too small to include GnRH-antagonist cycles in the study, which is also a major limitation.

In conclusion, we retrospectively analyzed the effect of Gn dose on embryo quality and clinical outcomes with AMH stratification in IVF cycles. We concluded that the total dose of Gn may have different effects on embryo quality at different serum AMH levels, and the negative effects of total dose of Gn on clinical outcomes may be realized by impairing both embryo quality and endometrium. To improve clinical outcomes, doctors should not only consider the possible negative effects of high-dose Gn doses on embryo quality and endometrium, but also the risk of OHSS. How to reduce the financial cost of IVF treatment cycle is also an important factor for doctors to consider. Using high-dose Gn in fresh cycles to obtain more embryos to be completely frozen and followed by frozen-thawed embryo transfer is still a good measure to reduce the risk of OHSS and adverse endometrial effects in fresh cycles. How to balance economic costs with improving clinical outcomes remains a challenge for physicians in practice. Consequently, the use of Gn should adhere to individualized principle and adopt the minimum dosage with expected therapeutic efficacy to reduce adverse effects on embryo quality and clinical outcomes.

### Supplementary Information

**Additional file 1: Table S1.** Cycle characteristics of different female age among the study population.

## Data Availability

The datasets used and/or analyzed during the current study are available from the corresponding author on reasonable request.

## Abbreviations

COS: Controlled ovarian stimulation
Gn: Gonadotropin
IVF-ET: In vitro fertilization-embryo transfer
OHSS: Ovarian hyperstimulation syndrome
PGT: Preimplantation genetic testing
FSH: Follicle stimulating hormone
ICSI: Intracytoplasmic sperm injection
GnRH: Gonadotropin releasing hormone
BMI: Body mass index
AMH: Anti-Müllerian hormone
LH: Luteinizing hormone
hCG: Human chorionic gonadotropin
