# Supplementary material for: Effects of total gonadotropin dose on embryo quality and clinical outcomes with AMH stratification in IVF cycles: a retrospective analysis of 12,588 patients
Source: Eur J Med Res. 2024 Mar 12;29:167. doi: 10.1186/s40001-024-01768-w (PMC10929233; doi:10.1186/s40001-024-01768-w)
Supplement: Supplementary file 1 — Additional file 1: Table S1. Cycle characteristics of different female age among the study population. [file 40001_2024_1768_MOESM1_ESM.docx]

| **Female age (years)** | **<35** | **35-42** | ***P* Value** |
| --- | --- | --- | --- |
| Cycles | 9683 | 2905 |  |
| Female BMI (kg/m^2^) | 21.31±1.99 | 21.91±1.82 | <0.001 |
| Serum AMH (ng/ml) | 3.45±1.97 | 2.28±1.56 | <0.001 |
| Basal FSH (mIU/ml) | 6.82±2.15 | 7.59±2.76 | <0.001 |
| Gn duration (days) | 13.23±1.90 | 13.43±2.04 | <0.001 |
| Total Gn dosage（IU） | 2294.37±934.79 | 3207.26±1055.99 | <0.001 |
| Number of oocytes retrieved | 14.56±6.69 | 10.68±6.10 | <0.001 |
| Number of MII | 11.91±5.94 | 8.79±5.30 | <0.001 |
| Normal fertilization rate (%) | 67.84 | 67.33 | 0.083 |
| Average number of ET | 1.70±0.46 | 1.69±0.46 | 0.728 |
| Stage of ET |  |  |  |
| day 3 (%) | 76.37 | 86.33 | <0.001 |
| day 5/6 (%) | 23.63 | 13.67 | <0.001 |
| Endometrial thickness on ET day | 12.52±2.64 | 12.10±2.89 | <0.001 |
| **Embryo quality** |  |  |  |
| Top-day3 embryo rate (%) | 67.67 | 65.82 | <0.001 |
| Blastocyst formation rate (%) | 57.22 | 46.91 | <0.001 |
| High-quality blastulation rate (%) | 20.59 | 15.02 | <0.001 |
| **Clinical outcome** |  |  |  |
| Implantation rate (%) | 51.95 | 30.54 | <0.001 |
| Clinical pregnancy rate (%) | 68.86 | 44.35 | <0.001 |
| Live birth rate (%) | 54.56 | 29.75 | <0.001 |

AMH, anti-Müllerian hormone; Gn, gonadotropin; BMI, body mass index; FSH, follicle-stimulating hormone; MII, mature oocytes; ET, embryo transfer. P < 0.05 indicates significant difference.

Additional Table 1. Cycle characteristics of different female age among the study population.
